# Neuropsychological performance in alcohol use disorder: a Portuguese study

**DOI:** 10.1192/j.eurpsy.2022.2156

**Published:** 2022-09-01

**Authors:** I. Faria, C. Silva, F. Sola, K. Ramos

**Affiliations:** Coimbra Hospital and University Centre, Psychiatry, Coimbra, Portugal

**Keywords:** Neuropsychological performance, cognitive functions, Alcohol use disorder

## Abstract

**Introduction:**

Alcohol consumption has devastating psychosocial and health consequences, with effects on cognitive functions. Recent studies have highlighted that patients with diagnosis of alcohol dependence syndrome have cognitive deficits in executive function, visuospatial ability, attention, procedural memory, verbal fluency and processing speed.

**Objectives:**

The aim of this study is to characterize the sociodemographic and clinical patterns of the study sample and the cognitive deterioration severity and type.

**Methods:**

A retrospective observational study was conducted with patients who had alcohol use disorder diagnosis at Dual Pathology Outpatient and Inpatient Unity, Psychiatry Department, at Coimbra Hospital and University Center, Portugal. Patients were admitted from 1/1/2016 and 30/09/2021, and submitted at neuropsychological structured evaluation. From the initial sample, major neurocognitive disorder, intellectual development disorder, cerebrovascular accident, traumatic brain injury and neurosurgery were excluded.

**Results:**

The results show significant cognitive impairment in executive function, memory, verbal fluency and visuospatial ability.

**Conclusions:**

Our results support the hypothesis of widespread impairment resulting from alcohol consumption. Cognitive impairment can limit the psychotherapeutic intervention, the adherence to pharmacological therapy and abstinence maintenance. The sheer presence of alcohol use disorder should encourage a neuropsychological evaluation. Further studies are needed in this area to prevent and outline an early intervention.

**Disclosure:**

No significant relationships.

